# Rapid degeneration and neurochemical plasticity of the lateral geniculate nucleus following lesions of the primary visual cortex in marmoset monkeys

**DOI:** 10.1016/j.crneur.2024.100141

**Published:** 2024-11-28

**Authors:** Gaoyuan Ma, Jonathan M. Chan, Katrina H. Worthy, Marcello G.P. Rosa, Nafiseh Atapour

**Affiliations:** Neuroscience Program, Biomedicine Discovery Institute and Department of Physiology, Monash University, Clayton, VIC, 3800, Australia

**Keywords:** Lateral geniculate nucleus, Striate cortex lesion, Retrograde degeneration, Blindsight, Primate, Calbindin

## Abstract

Lesions of the primary visual cortex (V1) cause retrograde neuronal degeneration, volume loss and neurochemical changes in the lateral geniculate nucleus (LGN). Here we characterised the timeline of these processes in adult marmoset monkeys, after various recovery times following unilateral V1 lesions. Observations in NeuN-stained sections obtained from animals with short recovery times (2, 3 or 14 days) showed that the volume and neuronal density in the LGN ipsilateral to the lesions were similar to those in the contralateral hemispheres. However, neuronal density in the lesion projection zone of LGN dropped rapidly thereafter, with approximately 50% of the population lost within a month post-lesion. This level of neuronal loss remained stable for over three years post-lesion. In comparison, shrinkage of the LGN volume progressed more gradually, not reaching a stable value until 6 months post lesion. We also determined the time course of the expression of the calcium-binding protein calbindin (CB) in magnocellular (M) and parvocellular (P) layer neurons, a form of neurochemical plasticity previously reported to be triggered by V1 lesions. We found that CB expression could be detected in surviving M and P neurons as early as two weeks after lesion, with the percentage of neurons showing this neurochemical phenotype gradually increasing over 6 months. Thus, neurochemical change precedes neuronal degeneration, suggesting it may be linked to a protective mechanism. This study highlights the limited time window for any possible interventions aimed at reducing secondary neuronal loss in the visual afferent pathways following damage to V1.

## Highlights

• Primary visual cortex (V1) lesion causes rapid thalamic degeneration and plasticity.
• Calbindin overexpression in lateral geniculate nucleus (LGN) precedes degeneration.
• Degeneration in LGN reaches to maximum after a month in marmoset monkeys.
• LGN volume shrinkage happens in a slower time scale of months after V1 lesion.

## Graphical abstract

**Figure:**
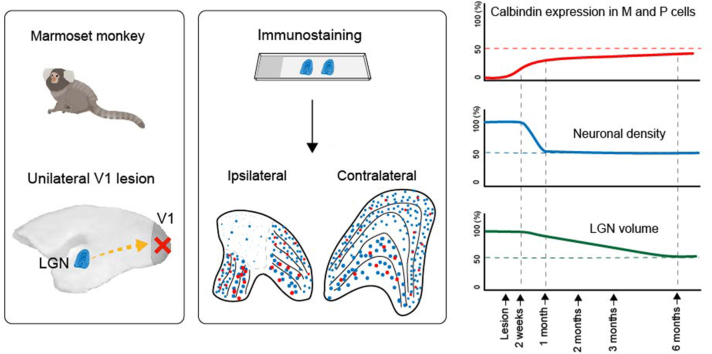

## 1 Introduction

Lesions in the primary visual cortex (V1) trigger degeneration and volume loss in the lateral geniculate nucleus (LGN), both in human and non-human primates (Polyak, 1957; Van Buren, 1963; Cowey and Stoerig, 1989; Hendrickson et al., 2015; Atapour et al., 2017, 2021; Simmen et al., 2022). The degeneration involves projection neurons in the magno- (M), parvo (P), and koniocellular (K) neurons, as well as corticogeniculate fibers (Wong-Riley, 1972; Atapour et al., 2017; Kinoshita et al., 2019). However, amount of degeneration may vary according to the species, lesion extent and survival time (Polyak, 1957; Van Buren, 1963; Mihailović et al., 1971; Atapour et al., 2017). V1 lesions in primates also cause cortical blindness, a condition that may reflect not only the immediate loss of cortical tissue, but also the secondary loss of LGN and retinal cells (Leopold, 2012).

Despite degeneration and volume loss caused by the lesion, there are a number of surviving neurons in the LGN, offering the potential for plasticity and recovery (Yu et al., 2018). One recently revealed form of plasticity are changes in protein expression in the surviving neurons (Atapour et al., 2021, 2022). Neurons in the normal LGN show specificity in their expression of calcium-binding proteins, with M and P neurons exclusively expressing parvalbumin (PV) and K neurons expressing calbindin-D28K (CB; Yan et al., 1996). In animals with long-term V1 lesions, this specificity is disrupted with many M and P neurons co-expressing CB and PV (Atapour et al., 2022). It has been found that some of the neurons undergoing this neurochemical change form projections to the middle temporal area (area MT) (Atapour et al., 2022), one of the key cortical areas hypothesized to mediate residual visual capacities following V1 lesions (Rosa et al., 2000; Ajina and Bridge, 2018). These findings reinforce the view that surviving LGN neurons may play a role in the residual visual abilities that remain within scotomas associated with V1 lesions.

Retrograde degeneration following V1 lesions is likely to be a gradual process (Mihailović et al., 1971; Wong-Riley, 1972; Cowey et al., 1999, 2011; Atapour et al., 2017). However, data on the exact pace of the degeneration remains sparse, particularly in the first weeks after V1 lesions. In addition, little is known about the time course of changes in calcium-binding protein expression. Here we address this knowledge gap by studying the cellular anatomy of the LGN in marmoset monkeys in the first weeks or months following V1 lesions. Marmosets are non-human primates which have been gaining prominence as a translational model for studies of human biology and disease (Solomon and Rosa, 2014; Mitchell and Leopold, 2015; Okano, 2021). Understanding the rate of progress of post-injury degenerative processes may provide important clues for future work aimed at minimising cell loss, and potentially greater preservation of residual visual abilities.

## 2 Materials and methods

### 2.1 Subjects

The present study was performed in a cohort of 21 marmoset monkeys (*Callithrix jacchus*), of which 14 received a unilateral V1 lesion in adulthood and 7 were non-lesioned controls. The animals were of either sex as detailed in Table 1. Eleven of these animals (4 lesioned, 7 controls) were part of other experiments involving fluorescent tracer injections (Majka et al., 2020, and unpublished observations) or electrophysiology experiments, not reported here. Their health condition and well-being were monitored throughout the experimental period. The experiments were conducted based on the Australian Code of Practice for the Care and Use of Animals for Scientific Purposes. All experiments were approved by the University Animal Ethics Experimentation Committee.

### 2.2 V1 lesions

The V1 lesion surgery was conducted following an updated procedure based on the technique introduced by Rosa et al. (2000). This procedure involves an occipital lobectomy along a vertical plane across the border between V1 and the second visual area (V2; Rosa et al., 1997), resulting in a complete loss of the representation of the visual field up to 10° eccentricity along the vertical meridian, and 20–30° along the horizontal meridian. Reconstructions of lesions and visual field defects created with this procedure have been reported previously (e.g. Yu et al., 2013, 2018; Atapour et al., 2021).

The animals were pre-medicated with oral meloxicam (Metacam; Boehringer Ingelheim, 0.1 mg/kg, i.m) and cephalexin (Ibilex; Alphapharm P/L, 30 mg/kg, i.m) 24 hours before the surgery. Atropine (Atrosite; Ilium, 0.2 mg/kg) was administered 30 minutes before anaesthesia, which was accomplished by inhalation of isoflurane (Isorrane; 4–5% in oxygen, Baxter). Dexamethasone (Dexason; Ilium, 0.3 mg/kg, i.m) was also administered. During the surgery, the animals were positioned in a modified stereotaxic head holder while their heart rates, body temperatures, and body oxygenation levels (PO_2_) were continually monitored. The anaesthetic condition was adjusted continuously (isoflurane, between 2 and 5%) to ensure the animals showed no spontaneous muscle activity and had no withdrawal reflexes. After craniotomy and durectomy the occipital pole was removed using a fine-tipped cautery, following a vertical excision along a plane extending from the dorsal surface of the occipital lobe to the cerebellar tentorium, across its entire mediolateral extent. After the removal of tissue, the resulting cavity was filled with haemostatic microspheres (Arista AH, BARD Davol Inc.) until the bleeding stopped. The surface of the wound was covered with ophthalmic film (Gelfilm, Pfizer Inc.), and the cavity was filled with Gelfoam (Pfizer Inc.). The skull flap was repositioned and secured with cyanoacrylate (Vetbond, 3M), followed by skin suture with polyglactin thread (5-0 Vicrly, Johnson & Johnson). The animals then were placed in an infant incubator (Atom Medical) for recovery and reintroduced to the home cage after recovery of mobility. During recovery, postoperative analgesia (oral meloxicam 0.05 mg/kg for adults, 3 d) and antibiotic (oral cephalexin 30 mg/kg, 5 d) was given. The animals demonstrated normal movement abilities including precise grasping, holding, jumping between branches, and obtaining food without assistance already on the day following surgery, and throughout the recovery period. With the exception of animals tested at very short survival times (<1 month), they were kept within family groups and lived in large cages with access to both indoor and outdoor environments. The short survival animals were kept in a facility that allowed close monitoring, in visual and auditory contact with other marmosets.

### 2.3 Tissue processing

Following variable survival periods (Table 1) the animals were anaesthetised with Alfaxan (Ibilex, 30mg/kg i.m.) and then overdosed with a pentobarbitone sodium injection (100 mg/kg i.v.). Following cardiac arrest, they were perfused with 0.1 M heparinised phosphate buffer (PB; pH 7.2) and 4% paraformaldehyde (PFA) in 0.1 M PB. The brain was removed and post-fixed for 24 hours in the same solution, after which cryoprotection was performed by immersion in 4% buffered PFA solutions with increasing concentrations of sucrose (10%, 20% and 30%). The brain was then snap-frozen and cut into 40 μm coronal sections using a cryostat (Leica, CM1850).

**Table 1:** Experimental details for all subjects.

Subject (Sex)	Survival (∼months)	Shrinkage ratio (% ipsi/intact)	Age at lesion (∼months)	Age at perfusion (∼months)	Analysed LGN
WA9(M)	39	50.84%	19	58	Both
WA14^a^(F)	31	52.30%	25	57	Both
WA13^a^(M)	28	68.61%	29	57	Both
WA16^b^(M)	23	57.36%	32	55	Both
WA15^a^(F)	12	43.17%	26	38	Both
WA25(F)	6	71.53%	26	32	Both
WA22(F)	3	85.11%	42	45	Both
WA23(F)	3	85.98%	37	40	Both
WA20(M)	2	75.87%	36	38	Both
WA21(F)	2	84.02%	44	46	Both
WA27(M)	1	78.83%	39	40	Both
WA32(F)	2 weeks	91.54%	30	30	Both
WA24(F)	3 days	96.65%	32	32	Both
WA26(M)	2 days	101.58%	45	45	Both
CJ227^a^(M)	–	102.51%	–	37	Both
CJ217^a^(M)	–	99.37%	–	34	Both
CJ200^a^(F)	–	98.39%	–	44	Both
F1741^a^(F)	–	–	–	42	Right
CJ174^b^(F)	–	–	–	32	Right
CJ170^b^(M)	–	–	–	29	Right
CJ167^b^(F)	–	–	–	28	Right

a Animals that received fluorescent tracer injections for other projects.

b Animals that participated in electrophysiology experiments for other projects. CJ217 and CJ227 were only used for volume analysis. (M) Male; (F) Female.

For NeuN staining, every fifth section was washed in a PB solution (0.1 M). The sections were incubated with a blocking solution (0.3% Triton-X100 and 10% horse serum in 0.1 PB) for 1 h at room temperature, and then treated with NeuN primary antibody (1:800, Merck Millipore, USA, MAB377, Clone A60, RRID: AB_2298772) for 46–48 h at 4 °C. This was followed by incubation with biotinylated horse anti-mouse IgG secondary antibody (1:200, PK-6102, Vectastain Elite ABC HRP kit, Vector Laboratories, USA, RRID: AB_2336821) for 30 min at room temperature. The sections were then incubated with ABC reagent (100μl of solution A and 100μl of solution B in 5 ml of 0.1M PB) for 30 min at room temperature. After incubation, sections were stained with DAB substrate working solution (DAB kit SK-4100, RRID: AB_2336382) for 30 min at room temperature, followed by three washes with PB (0.1M). The mounted sections were dried for 48 h before being coverslipped.

For fluorescence staining of calcium-binding proteins another series of sections (1 in 5) was incubated with blocking solution for 1 h at room temperature, followed by 46–48 h incubation in primary antibodies for CB (Calbindin-D28K; 1:50, Thermo Fisher Scientific, RRID: AB_2068509) and PV (1:500, Swant, code 235, RRID: AB_10000343). The secondary antibodies [1:600; Alexa Fluor® 488 (ab150109) and Alexa Fluor® 647 (ab150135)] were applied for 60 min at room temperature, followed by coverslip using antifade mounting medium (Vectashield, Vector Laboratories). These antibodies have been validated previously (Supplemental data, Atapour et al., 2022) and here we have provided further evidence in Extended Data Fig. 1.

**Fig. 1:**
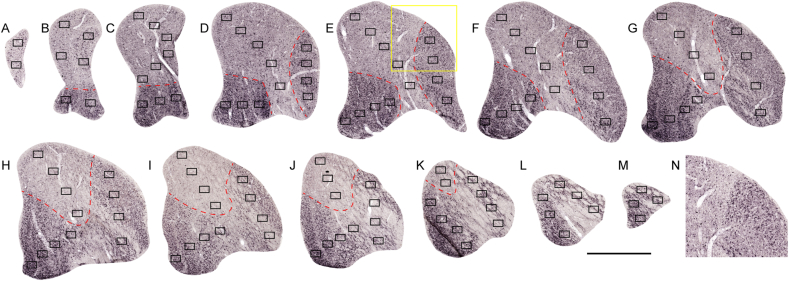
Design of sampling for stereological analysis. A–M: NeuN-stained coronal sections from WA25 covering the extent of lateral geniculate nucleus (LGN, interaural +3.15mm to +6.00mm, Paxinos et al., 2012), on which location and number of sampling frames (100 × 150 μm^2^) are shown. A represents the most posterior part of LGN. N: Inset in E. Red dashed lines separate the lesion projection zones (LPZ) from the rest of LGN. The boundaries of LPZ is determined according to cell size and the variation in neuronal density. The counting frames were placed in a radial dimension covering the different cellular layers in LGN. Evidence of no cellular staining in the absence of NeuN primary antibody is shown in the supplementary Fig. 1. Scale bar: 1mm. (For interpretation of the references to colour in this figure legend, the reader is referred to the Web version of this article.)

### 2.4 Quantification and statistical analysis

For NeuN-stained brain sections, slides were scanned with an Aperio Scanscope AT Turbo microscope (Leica Biosystems) at × 20 magnification under the resolution of 0.50 μm/pixel, and analysed using Aperio Image Scope software. Estimation of volume was done using Cavalieri estimator based on the area of LGN in equally spaced sections throughout the anteroposterior extent of this nucleus (Atapour et al., 2017; Gundersen and Jensen, 1987). For neuronal density, counting frames (150 × 100μm^2^) were placed systematically on each LGN section covering both degenerated and undegenerated sectors. In each section, 2–13 of counting frames were placed in a radial direction across all layers (Fig. 1). Only neurons with clear staining were counted, regardless of shape. Every neuron that was fully located inside the frame or that intersected the top or right edges was included. Data of all sections were combined for analysis. The cell counts were converted to densities (cells/mm^3^) by taking into consideration the section thickness and a shrinkage factor of 0.801 (Atapour et al., 2019).

The fluorescence immunostained sections were scanned using confocal microscopes (Nikon C1 invert, 20x magnification, filters 488, 647) and analysed with ImageJ software (Fiji, USA). The LGN was sampled in fixed sized square frames (638 × 638μm^2^) from the middle sections of the LGN, where the placement of the counting frames covered most of LGN (Atapour et al., 2021). Only neurons with clear fluorescent signal were counted using the cell counter plug-in. For analysis of CB expression in M and P neurons, we manually marked all PV-immunoreactive neurons within the M and P layers of LGN. We then counted CB-expressing neurons from the population of marked neurons, and calculated the percentage of double-labelled neurons. All statistical analysis was performed using Prism 9 (GraphPad software, USA). Data were analysed using student's t-test, one- or two-way ANOVA and linear regression where applicable and presented as mean ± standard error of the mean (SEM). Statistical results with a p value < 0.05 were considered statistically significant.

## 3 Results

### 3.1 V1 lesion triggers neuronal loss in the LGN within a month

**Fig. 2:**
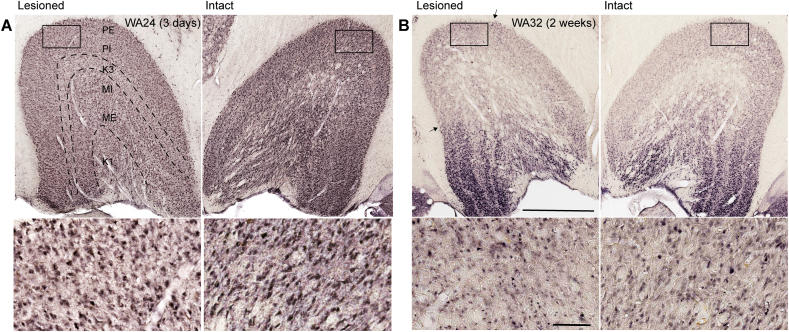
No visible cellular loss is observed in the lateral geniculate nucleus (LGN) three days (A) or two weeks (B) after lesion in the primary visual cortex (V1). Top: NeuN-stained LGN in the lesioned (left) or intact hemispheres (right). Dashed lines show layer boundaries. PE: parvocellular external layer, PI: parvocellular internal layer, K3: Koniocellular 3, MI: magnocellular internal layer, ME: magnocellular external layer, K1: Koniocellular 1. Scale bar: 1 mm. Black arrows point to the limits of degenerated zone in B. Bottom: Boxed areas in the expected lesion projection zone (parvocellular layers) and a corresponding region of the contralateral hemisphere are shown in higher magnification. Scale bar = 100μm.

**Fig. 3:**
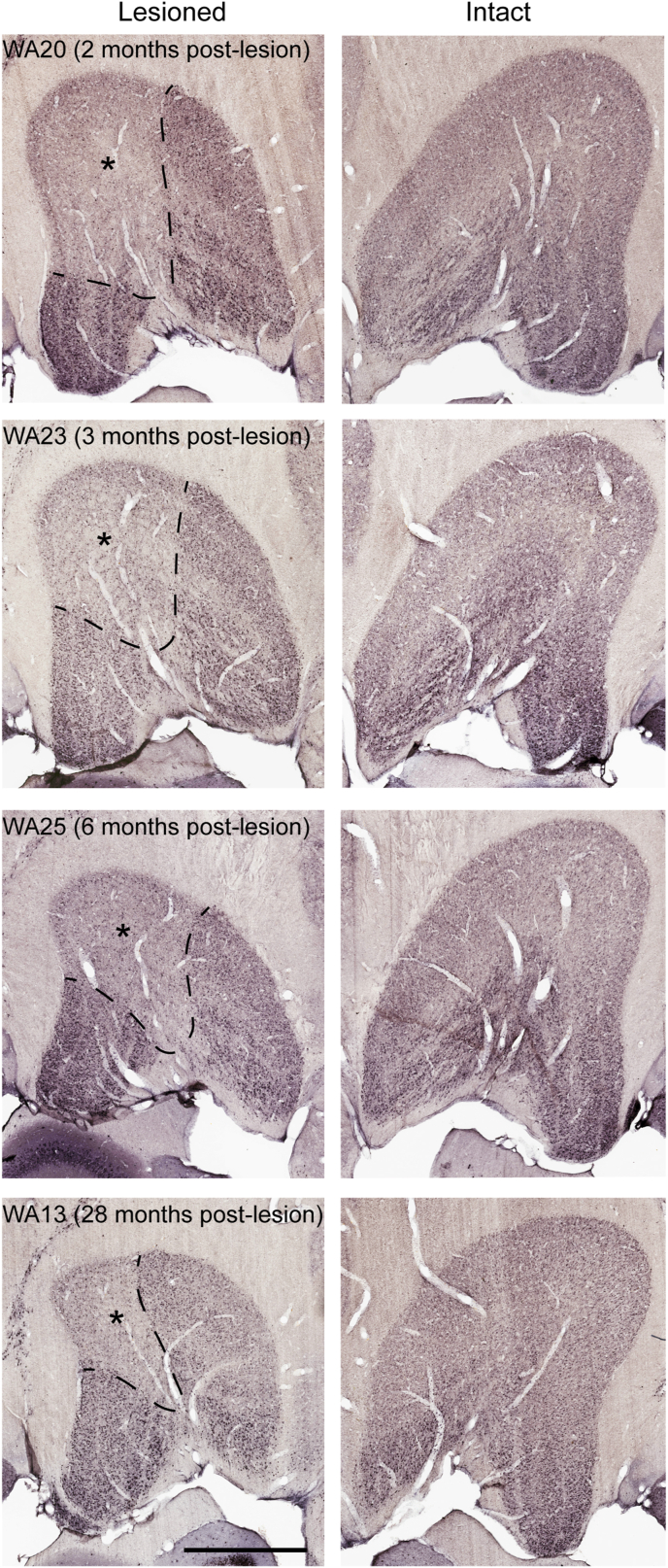
Lesions of the primary visual cortex (V1) trigger retrograde degeneration in the lateral geniculate nucleus (LGN). A: Representative images of NeuN-stained coronal sections through the LGN (interaural +4.80mm), obtained from four animals with different post-lesion survival times. In all cases the LGN ipsilateral to the V1 lesion shows a well-defined lesion projection zone (LPZ, identified by dashed line/asterisk), while the contralateral LGN shows normal lamination and shape. Scale bar: 1mm.

**Fig. 4:**
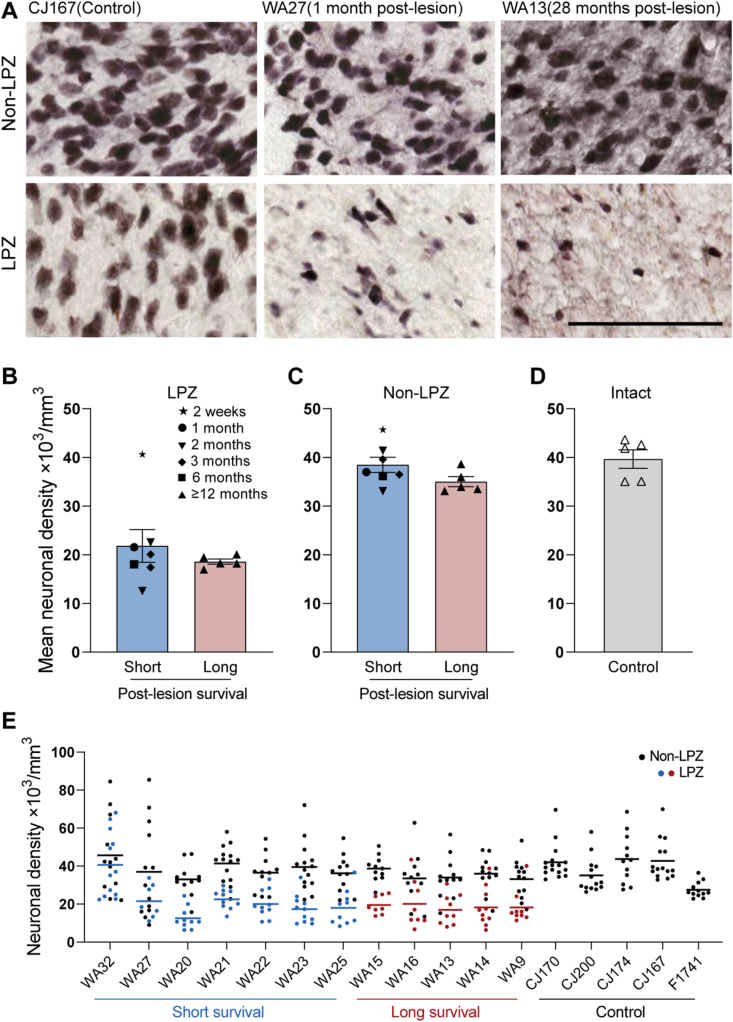
Lesions of the primary visual cortex (V1) cause significant neuronal loss in the lateral geniculate nucleus (LGN) within the first month. A: Representative images from the lesion projection zone (LPZ) and non-LPZ regions in control (CJ167), short survival (WA27) and long survival (WA13) cases. Scale bar = 100 μm. B–D: Mean ± SEM of neuronal density of LGN for the short and long survival animals in LPZ (B) and non-LPZ (C) as well as control animals (D). Different post-lesion survival times are identified with different symbols in the short survival group (two weeks to 6 months post lesion). Density values for the case of two weeks survival (WA32) are marked on A and B for comparison purposes, but they were not included in the averaging and statistical analysis. E: Variability of neuronal density across LGN layers for all cases. Each circle represents a sampling frame as depicted in Fig. 1. Small lines represent mean, also indicated in B-D.

In the animals perfused 2–3 days post lesion, NeuN staining of the LGN ipsilateral to the lesion revealed no changes in volume, lamination and neuronal size/density in comparison with the contralateral LGN (Fig. 2A). In all other cases (e.g. Fig. 3) a pale staining region corresponding to the lesion projection zone (LPZ; the sector of LGN that corresponds topographically to the V1 lesion) was obvious in the ipsilesional LGN, similar to previous observations (Kisvárday et al., 1991; Hendrickson et al., 2015; Atapour et al., 2017). Higher magnification views of neurons within and outside the LPZs are shown in Fig. 4A, showing that the pale staining regions were characterised by obvious reduction in neuronal density. In one animal (WA32), which survived only two weeks after V1 lesion, the LPZ was only partially evident, particularly at higher magnifications, by virtue of morphological changes such as reduced cell size and the loss of definition in the contours of neuronal nuclei (Fig. 2B). In this case, the volume of the LGN ipsilateral to the lesion was 8% lower than that on the unaffected side, which slightly surpasses the typical variability in LGN volume estimates between hemispheres, further indicating an ongoing degenerative process.

For quantitative analysis, the cohort of animals was divided into short survival (<6 months, excluding animals that only survived 2–3 days) and long survival (12–39 months) groups, the latter corresponding to time points already explored (Atapour et al., 2017, 2022; Chan et al., 2021). As shown in Fig. 4B, the neuronal density in the LPZs dropped to nearly half of the values observed outside the LPZs in the same animals, or in control animals. This drop was evident in all cases that survived at least 1-month post lesion, indicating a rapid neuronal loss following V1 lesion. In contrast, in the case with a 2-week survival the neuronal density in the LPZ was similar to the values observed outside the LPZ or in control animals (star in Fig. 4B), indicating that the apoptotic process had not been completed. This is despite the surviving neurons displaying more variable degrees of size, shape and definition compared to the healthy-looking neurons of the equivalent area in the contralateral LGN (Fig. 2B), suggesting initial stages of degeneration. Density values for this case are shown in Fig. 4 for comparison purposes, but were not included in the statistical analyses below.

The observed neuronal loss was similar among all cases in the short survival group (1–6 months survival, Fig. 4B). Comparison with those in the long survival group also did not reveal any differences for neuronal density in the LPZ, indicating no further degeneration [Fig. 4B and C, two-way ANOVA; survival time: *F* (1, 20) = 2.15, p = 0.16, zone: *F* (1, 20) = 52.71, p < 0.0001, interaction; *F* (1, 20) = 0.0027, p = 0.96, post hoc; LPZ vs. non-LPZ; short survival (21.81 ± 3.37 vs 38.49 ± 1.56 × 10^3^/mm^3^, p < 0.0001), long survival (18.59 ± 0.54 vs. 35.02 ± 1.03 × 10^3^/mm^3^, p < 0.001)]. The mean neuronal density outside the LPZ was similar to that found in the LGN of non-lesioned control animals, both in the short and long survival groups [Fig. 4C and D, One-way ANOVA, F (2, 14) = 0.49, p = 0.62), control; 39.65 ± 1.90 × 10^3^/mm^3^]. Neuronal densities acquired from all sampling frames in each LGN (as depicted in Fig. 1) has been shown in Fig. 4E, suggesting a variable neuronal density in different LGN layers, although this variability remained relatively similar across cases.

### 3.2 LGN shrinkage advanced with the time after V1 lesion

**Fig. 5:**
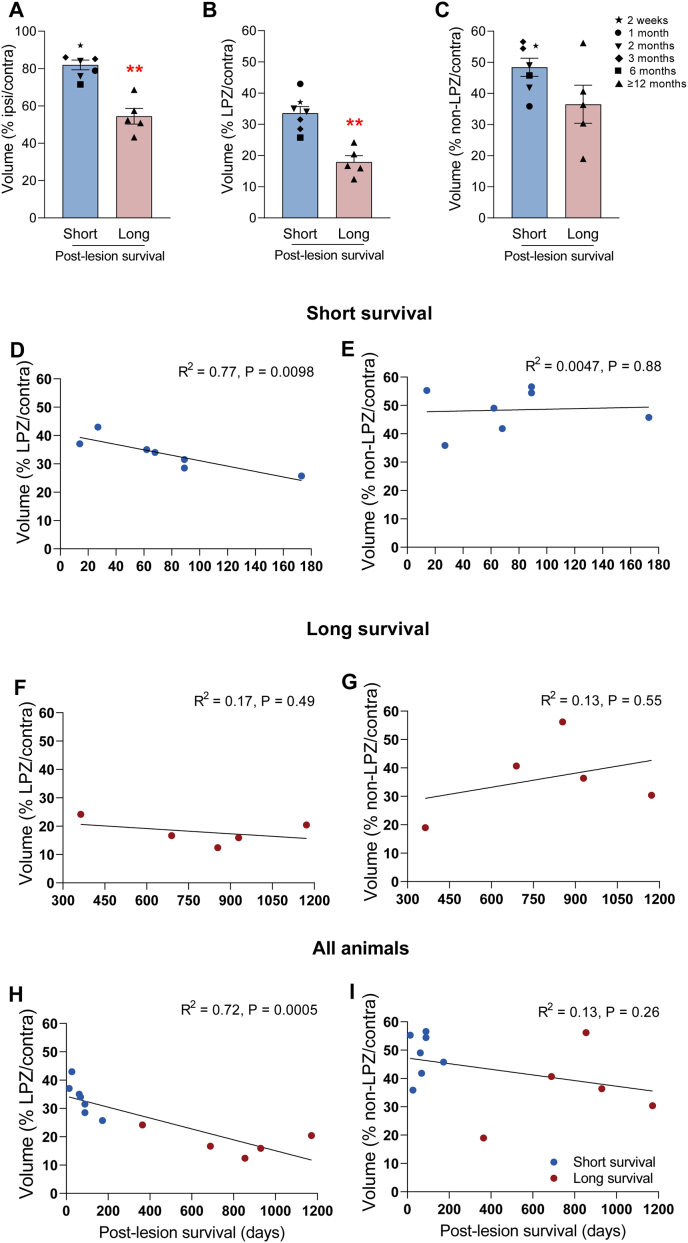
Lateral geniculate nucleus (LGN) volume loss correlates with the post-lesion survival time. A–C: Mean ± SEM of volume of the LGN for animals with short (2 weeks–6 months, n = 7) and long (12–39 months, n = 5) post-lesion survival period. Volume is presented as percentage of ipsilateral LGN (A) or its sectors, lesion projection zone (LPZ, B) and non-LPZ(C) compared to the contralateral LGN. Different post-lesion survival times are identified with variable symbols in the short survival cases. ∗∗p < 0.01. D–I: Percentage mean volume of LPZ (D, F, H) or non-LPZ (E, G, I) sectors are plotted against the post-lesion survival time. Data obtained in the short survival and long survival groups are shown separately (D–G) or combined (H–I). R2 represents the significance levels for the slopes of simple linear regressions for each condition.

The average LGN volume loss (ratio of ipsilesional LGN volume to contralesional LGN volume) was more extensive in the long survival group compared to the short survival group (Fig. 5A, *t*-test; long survival vs. short survival, 54.46 ± 4.21 vs. 81.95 ± 2.64%, p = 0.0025). To account for the possibility of variations in lesion sizes, we ran a separate analysis comparing the percentage loss of volume in the LPZ and non-LPZ regions, compared to the contralesional LGN volume. This analysis showed that the difference between the two groups originates mainly from changes in the LPZ volume (Fig. 5B; long vs. short survival, LPZ; 17.93 ± 2.02 vs. 33.55. ±2.15%, p = 0.0025). Although the analysis of non-LPZ volume shown in Fig. 5C indicates some variability across cases, the results did not reach statistical significance (non-LPZ; 36.52 ± 6.12 vs. 48.40 ± 2.919%, p = 0.15). In summary, the result indicates a significantly smaller volume loss in the short survival group.

To further understand the differences in volume loss in relation to survival times, we plotted percentage volume (i.e. the ratio of the volumes in the LPZ vs. contralesional LGN) against the survival time post-lesion in Fig. 5(D–I). A negative linear correlation was observed for LPZ volume (compared to contralesional LGN, Fig. 5H; linear correlation, R^2^ = 0.72, p = 0.0005). When the correlations were conducted separately for the short and long survival group, only the short survival group demonstrated a statistically significant negative correlation (linear correlation, LPZ; Fig. 5D; R^2^ = 0.77 p = 0.0098, 5F; R^2^ = 0.17 p = 0.49). This finding is consistent with the previous study that the shrinkage of LGN stabilised within six to seven months following V1 lesions (Atapour et al., 2017). The normalised volume of non-LPZ (as compared to contra-lesion LGN), on the other hand, did not show statistically significant changes in both short and long survival groups (linear correlation, non-LPZ; Fig. 5E; R^2^ = 0.0047, P = 0.88, 5G; R^2^ = 0.13, p = 0.55, 5I; R^2^ = 0.13, p = 0.26). Thus, the results indicate a substantial rapid loss of neurons occur first, followed by a gradual shrinkage of the LGN after lesion, and argue against differences in lesion extent being a confounding factor.

### 3.3 Calbindin positive M and P neurons exist as early as two weeks post lesion

**Fig. 6:**
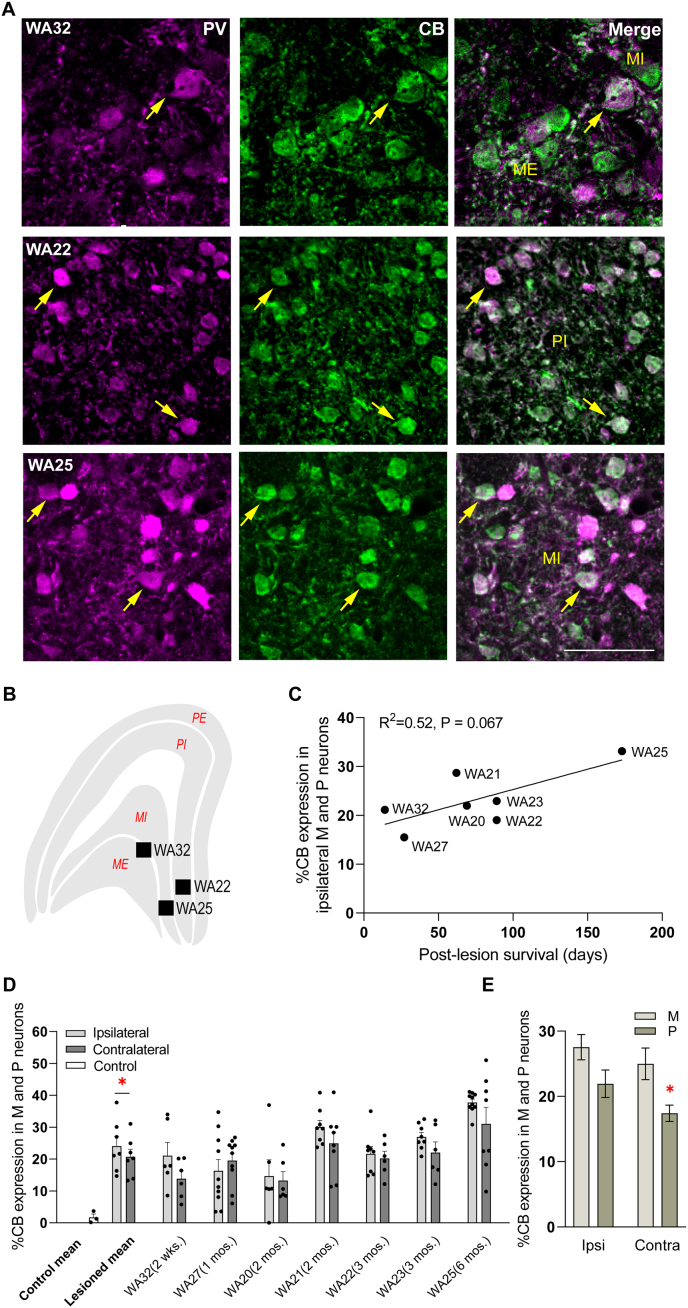
Calbindin (CB) immunoreactivity in parvalbumin (PV)-expressing neurons of lateral geniculate nucleus (LGN) is present after two weeks following lesions of the primary visual cortex (V1). A: Representative images showing CB expression in PV-expressing magnocellular (M) and parvocellular (P) neurons for 3 animals which survived two weeks (WA32), three (WA22) and six (WA25) months post-lesion. Yellow arrows point to individual neurons expressing both CB and PV. Scale bar: 100μm. B: Locations of individual images within the LGN. ME: magnocellular external layer, MI: magnocellular internal layer, PE: parvocellular external layer, PI: parvocellular internal layer. C: Mean percentage of CB expression in the ipsilesional M and P neurons is plotted against post-lesion survival time. R^2^ indicates the significance level for the slope of a simple linear regression. D: Percentage mean ± SEM of CB expression in M and P neurons of the LGN for control and lesioned cases (the latter shown as individual cases and also averaged). Black circles represent individual sections analysed. E: Averaged data from lesioned cases (shown in D) is indicated separately for M and P layers. ∗p < 0.05. Evidence of no cellular staining in the absence of primary antibodies for CB and PV is shown in Supplementary Fig. 1. (For interpretation of the references to colour in this figure legend, the reader is referred to the Web version of this article.)

Previous studies have demonstrated that, in addition to degeneration, V1 lesions elicit neurochemical changes in the LGN M and P neurons (Atapour et al., 2021, 2022). In particular, these neurons, which normally only express PV (Goodchild and Martin, 1998; Sincich et al., 2004; Atapour et al., 2022), also show co-expression of CB, including in M neurons projecting to area MT (Atapour et al., 2022). Here we explored the timing of CB expression in M and P neurons following V1 lesions. The CB positive neurons were quantified from populations of PV-expressing neurons (M and P) in the non-LPZ in the short survival group. This confirmed the presence of CB in M and P cells in all cases, ranging from 2 weeks to 6 months post-lesion (Fig. 6). Example images of CB expression in M and P neurons are shown in Fig. 6A–B. Regression analysis revealed a trend towards greater CB expression in M and P neurons with the survival time, although this did not reach a statistically significant level (Fig. 6C, R^2^ = 0.52 p = 0.067). While degeneration is exclusive to the ipsilesional LGN, a minimum of about 11% CB co-localisation with PV was also detected in the contralateral LGN (Fig. 6D), confirming that this neurochemical change triggered by the unilateral lesion occurs in both hemispheres (Atapour et al., 2022). The average values obtained from all lesioned animals showed that the proportion of CB expressing M and P neurons was significantly higher in the ipsilesional LGN (Fig. 6D, paired *t*-test; ipsi vs contra; 24.09 ± 3.07 vs. 20.71 ± 2.34, P = 0.039). Lack of CB expression in M and P cells in nonlesioned animals is indicated as an average of three cases in Fig. 6D. These data, obtained using the same methodology, have been published previously as individual cases (Atapour et al., 2022). Higher percentage of M neurons showed CB expression in comparison with P neurons only in the contralateral LGN [Fig. 6E; two-way ANOVA; interaction, F (1, 102) = 0.24, P = 0.62; hemisphere, F (1, 102) = 3.19, P = 0.077; Cell type, F (1, 102) = 11.03, P = 0.0012, post hoc: M vs. P: Ipsi; 27.54 ± 1.94 vs. 21.94 ± 2.09, P = 0.17, Contra: 24.98 ± 2.43 vs. 17.41 ± 1.24, P = 0.048].

## 4 Discussion

The present study quantified changes in the structure and neurochemistry of the marmoset monkey LGN across an extended time span following V1 lesions. Our study builds on the prior knowledge of degenerative changes in primate LGN, by employing quantitative, comprehensive assessment of neuronal density, volume and neurochemistry. Using specific methods for the labelling of neurons, we found that the vast majority of the changes in neuronal density in the LPZ occurred between 14 and 30 days post lesion, with no further changes up to 3 years post lesion. However, the shrinkage of the LPZ happened according to a slower timescale, not being completed until 6 months post lesion. In parallel to these degenerative changes, we found that CB expression could be detected in surviving M and P neurons as early as 2 weeks post lesion.

### 4.1 Neuronal loss in the LGN

V1 lesion induced fast retrograde degeneration, which resulted in a sharp decrease of neuronal density in the LGN within just 1 month of the lesions. The fast nature of degeneration is in agreement with previous studies in other primate species (Mihailović et al., 1971; Wong-Riley, 1972), which used the Nissl stain to assess neuronal loss. In squirrel monkeys, neuronal degeneration is reported to be completed within 42 days, with early signs of retrograde degeneration being evident only 5–8 days after lesions (Wong-Riley, 1972). This time course is compatible with that suggested by our data, where no cell loss was detected 3 days after lesion, while there were indications of changes in cell morphology and size after 2 weeks, possibly as signs of degenerative process. Despite changes in morphologies, our quantification suggests that there is no overt neuronal loss at this time point. The small variability in the estimates of neuronal density in the LPZ among cases with survival times of a month or more indicates that the degeneration process has been largely completed within a month, supporting previous data in macaque monkeys (Mihailović et al., 1971), who reported completion of degeneration within 3–6 weeks.

The present study, employing quantitative assessment of neuronal density with a specific neuronal marker, extends previous knowledge with additional significant details, over a wider time scale, and to a different primate species (marmoset). In the context of previous literature, these results are significant in two ways. First, they demonstrate that the time course of degeneration is similar despite large differences in brain size (and hence axonal length) among primate species. This species similarity indicates that a comparable time course is likely to apply to the human brain. Second, they identify a time window during which possible future interventions to reduce neuronal loss in the LGN need to apply. Long-term occipital lesions cause loss of function not only due to the direct loss of V1 cortex, but also subsequent degenerative changes in the LGN and retina (Cowey et al., 2011; Hendrickson et al., 2015; Atapour et al., 2017, 2021; Sepehrisadr et al., 2024). Preventing or minimising the secondary losses in the afferent visual pathway may be relevant for future therapeutic interventions aimed at retaining or improving residual vision.

Previous studies in macaque (Polyak, 1957; Van Buren, 1963; Mihailović et al., 1971) have suggested a potentially more significant cell loss in the LGN following V1 lesions, compared to observations in marmoset (Atapour et al., 2017, and present observations). In this context, the variability in lesion sizes, involving different parts of extrastriate visual cortex, can significantly contribute to variations in cell loss (Cowey et al., 1999). In the present study, the lesions consistently damaged the foveal and near-peripheral representations of V1 in one hemisphere, largely avoiding V2 except for near the boundary with V1 (for examples, see Rosa et al., 2000; Yu et al., 2013, 2018; Atapour et al., 2021). The difference may also be partially due to technical differences, including the use of Nissl staining in earlier studies, rather than the more specific NeuN stain, and, in some cases, qualitative assessment (Polyak, 1957; Van Buren, 1963; Mihailović et al., 1971). The possibility also exists that there are differences in the pattern of projections from LGN neurons to extrastriate areas between marmosets, macaques and humans, which could affect neuronal survival by allowing preservation of different numbers of synaptic targets outside the lesioned zones. However, currently there is no evidence of such differences (Yukie and Iwai, 1981; Bullier and Kennedy, 1983; Dick et al., 1991; Kaske et al., 1991; Sincich et al., 2004; Atapour et al., 2022; Ding, 2024), warranting further studies. Our observation of a variable distribution of neuronal loss across LGN (Fig. 4E), is partly reflective of the natural variability as we have reported previously (Atapour et al., 2017), however it may also reflect differential neuronal loss across LGN layers that requires further studies.

### 4.2 Loss of LGN volume

Atrophy of the LGN is a critical pathological indication for V1 lesions which can be quantified with non-invasive brain imaging (Simmen et al., 2022). Whereas volume reduction is clearly influenced by neuronal loss, these two aspects of degeneration can occur independently. For example, volume changes without concomitant loss of neuronal cell bodies have been observed in the pulvinar complex following V1 lesions, an observation that has been interpreted as indicating that volume reduction was likely due to changes in the neuropil (Chan et al., 2021).

In the present study, the volume loss in the LGN ipsilateral to the lesion increased with the survival time, despite the level of neuronal loss being similar across all cases. This observation was normalised for the lesion size (a factor which is likely to affect LGN shrinkage) by separate estimation of percentage volume loss for LPZ and non-LPZ. This correlation with survival time was not observed among long survival animals. This is consistent with previous observations (Atapour et al., 2017) that volume loss in the marmoset LGN does not progress beyond 6–7 months post lesion.

The delayed time scale for volume loss, compared to neuronal degeneration, may be due to the time required for debris removal (Westman et al., 2020; Liu et al., 2022), neuropil loss, or even remodelling of the capillary bed due to reduced energy requirements following apoptosis of LGN neurons. Moreover, cortico-geniculate axons and terminals fill much of the space between LGN neurons (Briggs and Usrey, 2011; Ichida et al., 2014), and their loss may also contribute to volume changes. Although evidence suggests that the time course and magnitude of retrograde and Wallerian (anterograde) degeneration are similar (Kanamori et al., 2012), there may be differences due to the differential site of injury along the axons (Lubińska, 1982) or heterogeneity of axons and their compartmentalisation (Conforti et al., 2007). Although it has been reported that early signs of cortico-geniculate terminal degeneration precede the retrograde degeneration of LGN neurons in squirrel monkeys (Wong-Riley, 1972), the biological complexity of degenerative processes (Conforti et al., 2007) may create difficulty in pinpointing the precise timing of events. What is clear is that the physical collapse of LGN volume is a more prolonged process than neuronal death.

### 4.3 Expression of CB in surviving neurons

Recent studies have shown that neurochemical changes are part of long-term plastic changes in the primate LGN following V1 injury (Atapour et al., 2021, 2022). Under normal conditions neurons in the M and P layers only express PV (Goodchild and Martin, 1998; Sincich et al., 2004; Atapour et al., 2022). Following V1 lesions they also co-express CB, a fact that we find evident even two weeks after lesion. However, the percentage of M and P neurons expressing CB following short survival times is lower (17–27%) than that observed after longer survival times (35–75%, Atapour et al., 2022), further emphasising the gradual nature of the emergence of CB expression in M and P neurons.

The present observations are in line with previous data that indicate changes in the neurochemistry of M and P neurons also occur in the LGN of the hemisphere contralateral to the lesion (Atapour et al., 2022). It has been suggested that V1 lesions could lead to reduced cortical feedback onto surviving LGN neurons (Briggs and Usrey, 2011), creating imbalanced activation of these neurons by the retina, which in turn would trigger CB expression (Gonzalez et al., 2005). Underpinning this assumption, M neurons, with the most significant CB overexpression after V1 lesions (Atapour et al., 2022 and present data), have larger than normal receptive fields (Yu et al., 2018) and the least degeneration of their retinal afferents (Cowey et al., 1989, 2011; Weller and Kaas, 1989; Hendrickson et al., 2015). Moreover, the more prevalent CB expression in M cells may suggest their higher potential to form new connections to the cortex (Atapour et al., 2022). CB expression has been highlighted as a protective mechanism linked to neuronal resilience under stress (Ng et al., 1996). Thus, concomitant with the ongoing degeneration in LPZ, the neurons in non-LPZ undergo neurochemical changes that may prepare them for better survival, or regenerative processes (Kim et al., 2006; Zupanc and Zupanc, 2006; Choi et al., 2008). Indeed, our observations are consistent with this hypothesis, as alterations in neurochemistry occur prior to the onset of neuronal loss. As recently demonstrated, the neurochemical change involves remodelling of the projections of M neurons, which form projections to extrastriate cortex (Atapour et al., 2022). Whether the emergence of the CB expression in M/P neurons within the first few months after lesion coincides with the process of remodelling their projection to extrastriate areas remains to be tested.

Changes in the neurochemistry of projection neurons, including expression of GABA (Atapour et al., 2021), might lead to better survival rates. We have also previously shown that after adult V1 lesions the percentage of GABAergic neurons is increased to almost sevenfold in the LPZ of LGN, suggesting that the cell loss due to retrograde degeneration is more prominent among projection neurons (Atapour et al., 2021). The progressive CB expression among projection neurons after lesion suggests that other neurochemical changes may also be linked with survival time, and thus a differential ratio of surviving excitatory neurons to interneurons in the LPZ is likely to happen over time.

## 5 Limitations and suggestions for future studies

The present study focuses on the changes occurring in LGN following V1 lesions in young adult marmosets. However, there is evidence that the age at which injury happens could affect the extent and pace of the degeneration process (Dineen and Hendrickson, 1981; Weller and Kaas, 1989; Hendrickson et al., 2015; Atapour et al., 2017). Thus, additional work focused on different stages of life is advisable in order to obtain a full picture of the effects of V1 lesions. Earlier work comparing lesions in young (2–6 weeks postnatal) and adult marmosets revealed modest physiological differences in the surviving LGN neurons (Yu et al., 2018).

The extent of the damage to the extrastriate visual cortex can also influence estimates of degeneration (Cowey et al., 1999). In the present experiments, the lesions were designed to be largely contained to V1, although involvement of parts of the second visual area near the border is inevitable. More extensive lesions resulting in damage to rostral extrastriate areas would likely result in more extensive loss due to the removal of projection targets of neurons that would otherwise survive the degeneration process. However, the few surviving neurons in the LGN continue to receive retinal projections even following complete unilateral hemispherectomies (Boire et al., 2001). Whether the surviving neurons following larger lesions undergo biochemical changes remains an open question. In the present study, none of the animals showed damage extending to rostral extrastriate areas such as the homologues of V4, or MT (Rosa and Elston, 1998; Rosa and Tweedale, 2000)

## 6 Conclusions

Our study demonstrates that the neuronal loss in the LGN proceeds rapidly in the marmoset, being largely completed within one month of V1 lesions, in agreement with previous work in other non-human primates. These findings support the view that the marmoset is a valuable animal model to study the anatomical and physiological plasticity processes involved in the aftermath of V1 lesions (Hagan et al., 2017). At the same time, the results indicate that the volume loss and biochemical changes in the LGN are slower processes, highlighting the complexity of the processes triggered by such lesions. This information has broader implications for our understanding of traumatic brain injury and future efforts towards minimising visual loss, and promoting regenerative processes which may improve the quality of residual vision in primates, including humans.

## Declaration of generative AI in scientific writing

Generative AI were not used in scientific writing of this manuscript.

## Funding sources

Funding was provided by the National Health and Medical Research Council to Marcello G. P. Rosa (APP1194206) and Nafiseh Atapour (APP2019011).

## Declaration of competing interest

The authors declare that they have no known competing financial interests or personal relationships that could have appeared to influence the work reported in this paper.

## Data Availability

Data will be made available on request.
