# Supplementary material for: Rapid degeneration and neurochemical plasticity of the lateral geniculate nucleus following lesions of the primary visual cortex in marmoset monkeys
Source: Curr Res Neurobiol. 2024 Nov 28;8:100141. doi: 10.1016/j.crneur.2024.100141 (PMC11697716; doi:10.1016/j.crneur.2024.100141)
Supplement: Multimedia component 1 [file mmc1.docx]

**
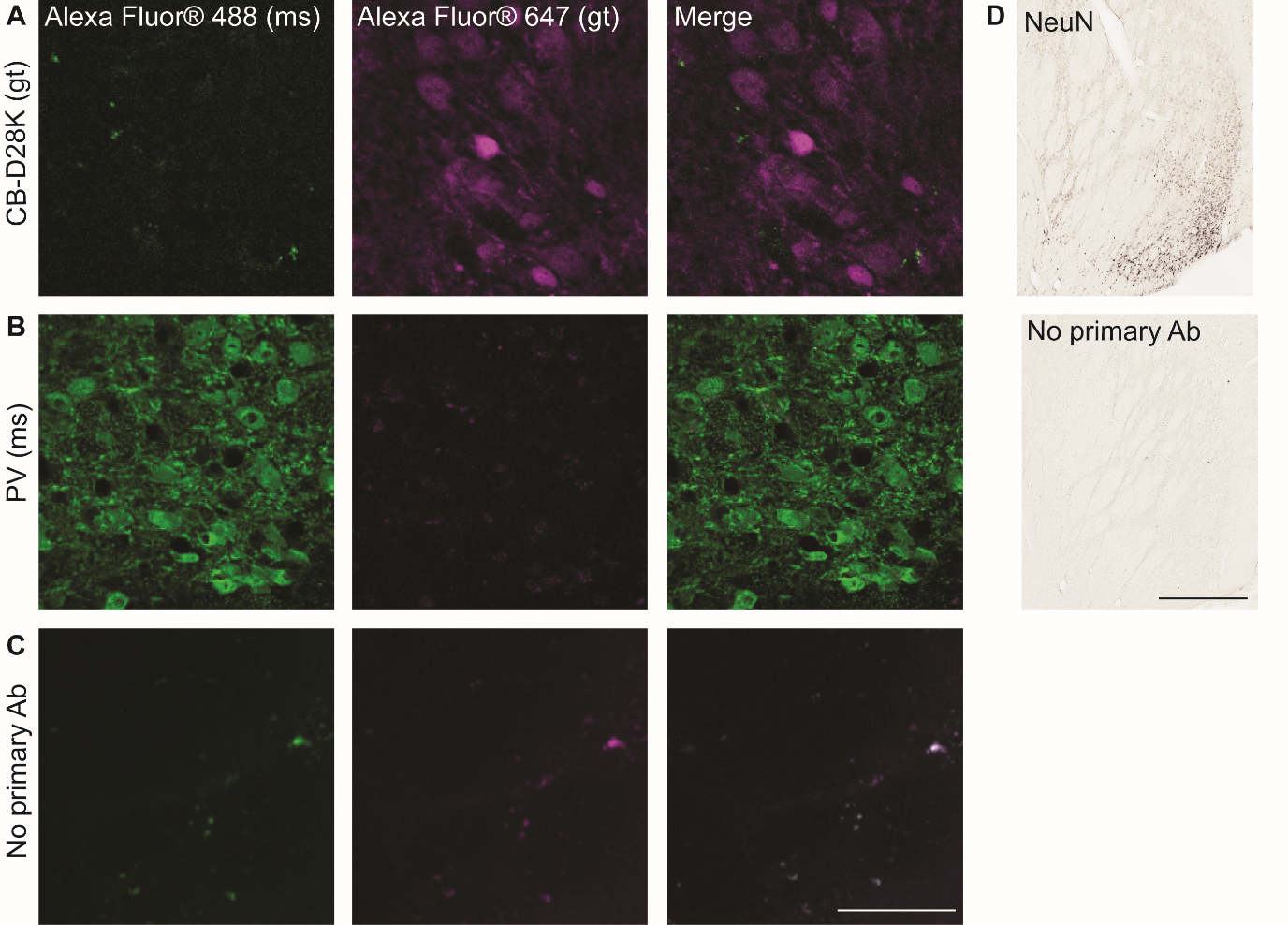
**

**Supplementary Figure 1.** Evidence of no cellular staining in the absence of primary antibody (Ab) in the lateral geniculate nucleus. A-C) Secondary fluorescent antibodies, anti-mouse (ms) Alexa Fluor® 488 and anti-goat (gt) Alexa Fluor® 647 that were used for visualisation of parvalbumin (PV)-positive and calbindin (CB)- positive neurons, respectively, did not produce any cellular staining when used without the application of the relevant primary antibody. Immunofluorescence staining for CB- D28K (RRID: AB_2068509, raised in gt) or PV (AB_10000343, raised in ms) was observed only when both primary and relevant secondary antibodies were present. D) Immunohistochemistry with (top) and without (bottom) the primary antibody against NeuN (RRID: AB_2298772), using non-fluorescent secondary antibody (Cat# PK-6102) followed by DAB (Cat# SK-4100). Scale 100 µm for (A-C) and 500 µm for (D).
